# Clinical Lectures on the Surgical Diseases of the Urinary Organs

**Published:** 1909-12

**Authors:** 


**Clinical Lectures on the Surgical Diseases of the Urinary Organs.** By P. J. FREYER, M.D., M.Ch. Pp. viii, 425. London: Baillière, Tindall & Cox. 1908.

The author states that his aim has been to give in these lectures a concise and practical *résumé* of the most recent knowledge of the subjects dealt with, and that the methods of treatment advocated are those which he has found to be the best by practical experience.

In the above aim he has, in our opinion, succeeded admirably. Many of the lectures are models of lucidity and full of sound advice, and will serve excellently for guidance in the conduct of treatment for stricture of the urethra, stone in the bladder, and senile enlargement of the prostate. What surgeon can fail to be interested in the matured presentment of the author's technique in prostatectomy ?

Much of the subject-matter has previously appeared in print, and is familiar to those in active surgical practice ; but the matter is worthy of re-perusal, and we are glad to have it collected in one handy volume.

The student will not find here a full account of cystoscopy nor of the various methods of differentiating the urine from each kidney. These subjects, it is true, are more or less discussed or alluded to, but they are not dealt with in the detailed manner of the sections devoted to enlarged prostate and stone in the bladder.

The treatment of chronic prostatitis by massage of the gland is stigmatised as disgusting, useless, and open to grave objection. The word " disgusting " might well have been omitted. Examination of the rectum is disgusting. Loss rather than gain results from dwelling upon the unæsthetic aspects of many necessary surgical procedures. We ourselves hold that massage of the prostate has a distinct field of usefulness.

